# Biomorphic Transformations: A Leap Forward in Getting Nanostructured 3-D Bioceramics

**DOI:** 10.3389/fchem.2021.728907

**Published:** 2021-09-07

**Authors:** Simone Sprio, Andrea Ruffini, Anna Tampieri

**Affiliations:** Institute of Science and Technology for Ceramics, Italian National Research Council, Faenza, Italy

**Keywords:** biomorphic transformation, heterogeneous chemistry, bone scaffold, bioactivity, apatites, bone regeneration, damage-tolerant behavior, biomimicry

## Abstract

Obtaining 3-D inorganic devices with designed chemical composition, complex geometry, hierarchic structure and effective mechanical performance is a major scientific goal, still prevented by insurmountable technological limitations. With particular respect to the biomedical field, there is a lack in solutions ensuring the regeneration of long, load-bearing bone segments such as the ones of limbs, due to the still unmet goal of converging, in a unique device, bioactive chemical composition, multi-scale cell-conducive porosity and a hierarchically organized architecture capable of bearing and managing complex mechanical loads in a unique 3D implant. An emerging, but still very poorly explored approach in this respect, is given by biomorphic transformation processes, aimed at converting natural structures into functional 3D inorganic constructs with smart mechanical performance. Recent studies highlighted the use of heterogeneous gas-solid reactions as a valuable approach to obtain effective transformation of natural woods into hierarchically structured apatitic bone scaffolds. In this light, the present review illustrates critical aspects related to the application of such heterogeneous reactions when occurring in the 3D state, showing the relevance of a thorough kinetic control to achieve controlled phase transformations while maintaining the multi-scale architecture and the outstanding mechanical performance of the starting natural structure. These first results encourage the further investigation towards the biologic structures optimized by nature along the ages and then the development of biomorphic transformations as a radically new approach to enable a technological breakthrough in various research fields and opening to still unexplored industrial applications.

## Introduction

Despite great progress in science and technology occurred in the past decades, to date insurmountable barriers still prevent the solution of crucial clinical needs, therefore a substantial quantum leap in technological development is highly desired. In this respect, we are probably witnessing the dawn of a new era. As the last decades were characterized by the extensive development and use of plastics and other synthetic disposable products, today the scientific community is intensively called to seek for new virtuous pathways. Particularly, scientists are turning their attention to nature, attracted by the unique structures of living beings, characterized by unusual and often contrasting properties exhibited at the same time, such as lightness and resistance, toughness and resilience, etc. Particularly, plants, shells, mammal bones and exoskeletons, show outstanding performances in terms of strength, compliance to multi-axial forces and self-repair ability, permitted by their hierarchical architecture organized along multiple scales from the nano-to the macroscopic size (Reznikov et al., 2018; Mishnaevsky and Tsapatsis, 2016). Such unique features were developed along a complex and million years-long evolutionary pathway, but often they are unachievable with current manufacturing technologies (Abdulhameed et al., 2019; Behera et al., 2021). Therefore, scientists are now intensively looking at radically new approaches permitting to copy and translate the unique and outstanding abilities of natural structures into new functional devices (Fish, 2020; Baines et al., 2020; Fish et al., 2021; Collier, 2013; Singh et al., 2019; Huang et al., 2019).

In this respect, it is important to consider that relevant functionalities of materials are strongly related to their chemical composition and their organized structure, both factors co-existing in a balanced equilibrium. Many applications, such as in energy production, photonics and biology, require functional phases showing specific nuances in the atomic composition and crystalline structure (Wongmaneerung et al., 2009; Qian et al., 2013; Tampieri et al., 2011; Limonov and De La Rue, 2016). The maintenance of chemical composition and nanostructure in 3D materials requires a conceptual change in the fabrication approach aimed at achieving smart performances and surpassing the basic paradigm in material science, particularly in the case of ceramic technology so far defined by powder processing, 3D forming and sintering.

Recent studies have highlighted alternative approaches, based on chemically-guided assembly processes as in the case of geopolymers (Provis and Bernal, 2014), which are natural materials with pozzolanic activity of great prospect for building applications, or of metastable calcium phosphatic compounds, able to consolidate at body temperature thanks to activation of dissolution/reprecipitation processes, thus functioning as bone cements (Schumacher and Gelinsky, 2015; Zhang et al., 2014). However, even if these approaches can generate 3D consolidated ceramics maintaining the nanostructure and reactive chemical composition, they are based on the assembly of building blocks such as powders or nanocrystals (Wegst et al., 2015). Such assembling phenomena are however difficult to be controlled and directed towards the achievement of constructs exhibiting structural organization and hierarchy so that, in spite of good strength, the mechanical performances are usually insufficient to bear relevant mechanical loads.

Pursuing the development of new 3D inorganic materials with a greater degree of control over the chemical composition, structural organization and mechanical performance, recent approaches have identified the possibility to use existing natural structures as models guiding the transformation processes generating *biomorphic* products (Wegst et al., 2015; Tan and Saltzman, 2004; Xie et al., 2019). A key aspect of such biomorphic transformation processes consists in the application of heterogeneous reactions that begin at the surface of the solid model and then propagate in its 3-D bulk structure by diffusive phenomena until chemical conversion is obtained overall. This approach entails the interaction of multiple physico-chemical parameters relevant during the biomorphic transformation, revolving around a fundamental aspect which is the kinetic control of the involved reactions, in turn being crucial to ensure: i) the attainment of the designed chemical composition, ii) the maintenance of the nanostructure as well as the hierarchical architecture leading to functional and mechanical competence.

## Need of a Leap Forward in Biomaterials Science to Respond to Unmet Clinical Needs

Biomorphic transformations can be extremely relevant to achieve new effective solutions for regenerative medicine. In fact, biomaterials play a key role as implants or scaffolds, intended to guide the endogenous cells to regrow and regenerate missing or diseased tissues, when spontaneous regeneration is prevented (Sprio et al., 2013). Such a problem is particularly crucial in orthopaedics, where the regeneration of critical size bone defects (i.e. defects that cannot heal spontaneously) requires the use of scaffolds bridging the defect and able to promote and sustain the appropriate cascade of biologic phenomena yielding the bone regeneration. However, the goal to obtain such regenerative scaffolds is still largely unmatched because of insuperable obstacles so far encountered in achieving the relevant *ensemble* of biomimetic physicochemical, structural and mechanical properties, able to appropriately drive and modulate the cell fate and metabolism.

Indeed, the bone tissue metabolism is mainly regulated by its mineral component: a nearly amorphous apatitic phase enriched with various biologically relevant ions in dynamic chemical equilibrium with the physiological environment, thus behaving like a “*living inorganic crystal*.” Seeking to mimic the chemical composition of bone, nano-apatites with such properties can be quite easily obtained by wet synthesis methods, but only in form of powders (Iafisco et al., 2014; Sprio et al., 2008). Conversely, the attainment of 3D apatitic scaffolds with effective regenerative ability was so far prevented by the impossibility to synthesize such a bioactive phase in the form of large consolidated bodies with multi-scale hierarchic structure, due to the need of high temperature sintering processes that invariably degrade the chemistry, nanostructure and pore architecture of the scaffold, as a whole responsible of its regenerative ability. The lack of these features limits the extent of those appropriate physicochemical and topotactic signalling to cells, promoting and sustaining the complex metabolic activity related to deposition and remodelling of newly formed bone. Besides the chemical signalling, cell mechanotransduction is also a biologically relevant phenomenon based on the conversion of mechanical forces into biochemical processes active at the cell level, by which osteoblasts continuously remodel the bone tissue structure to adapt to ever changing mechanical forces, thus permitting its self-repair in the case of damage of limited entity. Such phenomena are activated by the unique multi-scale hierarchical structure of the bone tissue that permits effective distribution of mechanical forces from the macro to the microscale and down to bone cells (Ingber, 1993; Pavalko et al., 2003). This mechanism is particularly crucial when load-bearing bones such as the ones of the limbs are involved. Today, the need of restoring the mechanical functionality in such critical bony districts forces to adopt very invasive and poorly resolutive approaches based on the use of Ilizarov implants or, alternatively, metallic plaques and bank bone pieces that do not help bone regeneration and can, instead, provoke infections and bad clinical outcomes (Kanakaris and Giannoudis, 2007; Longo et al., 2012; Patil and Montgomery, 2006).

Even when using sinter-free methods to consolidate apatitic phases into porous 3D devices, as obtained with recently developed apatitic bone cements, the lack of multi-scale open and interconnected porosity organized in a hierarchical architecture hampers effective vascularization of the whole scaffold and to mimic the outstanding mechanical ability of the natural bone (Roffi et al., 2017; Rho et al., 1998). Furthermore, in such a kind of materials the low temperature consolidation mechanism, yielding physical entanglement of acicular apatitic particles, does not produce a cohesion between the ceramic grains, sufficient to ensure adequate biomechanical performance.

Seeking to a paradigmatic change in bioceramics development, we noticed that natural vegetal structures show extraordinarily complex architectures organized along multi-scale hierarchies, capable of conferring outstanding mechanical performance, lightness and self-repair capacity, similarly as shown by the bone structure. Such vegetal architectures are therefore ideal models that can inspire the design of new generation biomorphic bone scaffolds with superior and unpreceded functional properties. The first pioneering attempts to generate 3D inorganic materials through transformation of natural woods into oxides or carbides date from the early 2000s (Li et al., 2006; Rambo and Sieber, 2005; Sieber et al., 2000; Greil, 2001; Esposito et al., 2004). Following, biomorphic inorganic structures for application as bone substitutes were obtained by transformation of natural woods into biocompatible silicon carbide scaffold, suitable as inert device to simply fill and repair the bone defect. In such experiments, pyrolysis processes were used to convert the natural wood structure into a carbon template, then subjected to infiltration/reaction with molten silicon at high temperature thus activating the reaction Si-C forming silicon carbide (SiC) phase (Parfen’eva et al., 2005; González et al., 2003). Such a biomorphic SiC scaffold showed high mechanical strength permitting implantation in long bone defects in sheep (Filardo et al., 2020; Filardo et al., 2013). However, despite its biocompatibility SiC is a bio-inert material, thus with very limited ability to induce new bone formation and vascularization in the scaffold’s pores and, besides, it is unable to be resorbed by metabolic cell activity. Such a bio-inert scaffold is destined to remain unmodified within the bone defect and so the physical and mechanical discontinuity existing between the scaffold and the surrounding bone easily yield stress shielding effects leading to bone resorption with time and the increasing risk of new fractures (Navarro et al., 2008; Best et al., 2008).

In the attempt to obtain bone scaffolds with bioactive composition, previous studies developed biomorphic transformation processes to achieve calcium carbonate or calcium phosphate constructs. In one case, natural cork wastes were pyrolyzed, then infiltrated with a suspension of calcium salts, and finally sintered to achieve porous calcium carbonate bodies (Scalera et al., 2020). In a different study, a pyrolysed wood was infiltrated with calcium phosphatic sol-gel suspensions, then sintered to achieve biomorphic calcium phosphate scaffolds (Eichenseer et al., 2010). In both cases, the obtained scaffolds resulted in poor reproduction of the original natural structure and very low mechanical properties. These adverse effects can be ascribed to the use of liquid reactants. On one hand, ceramic suspensions usually exhibit viscosity preventing penetration into small, micron-size pores, thus hampering to achieve a precise replica of the original template structure. On the other hand, in ceramic suspensions, particularly when featuring low viscosity, the concentration of the ceramic powder/granules is often not sufficient to activate the grain coalescence process during sintering, as required to give mechanical properties to the final ceramic body.

In the attempt to overcome such limitations and obtain mechanically effective biomorphic scaffolds, suitable to fit critical size bone defects in load-bearing regions, we recently described the biomorphic transformation of rattan wood by using a multi-step process based on gas-solid reactions (Tampieri et al., 2019). The use of gaseous reactants was preferred in order to facilitate the chemical interaction with the solid template, in turn permitting a more accurate control of the reaction kinetics and of diffusive phenomena yielding phase transformation throughout the whole solid. The choice of rattan wood as a model of bone tissue was related to its structure, where wide channels (∼300–500 μm) are hierarchically interconnected with smaller pores thus forming a vascular network closely resembling the anisotropic osteon architecture typical of the compact bone. Such a structure inducing anisotropic mechanical properties and high vascularization capability, is particularly relevant in long, load-bearing bone segments.

The present review will describe chemical aspects inherent to such a biomorphic transformation process. It will be given an insight to main strategies adopted in the various steps to modulate the kinetic of concomitant chemical reactions occurring at the gas-solid interface and to limit the grain growth. This latter was relevant to achieve reactive intermediate products and facilitate the attainment of the final biomorphic scaffold. Furthermore, we show how the fine tuning of the reaction kinetics permitted to modulate the chemical composition and nanostructure of the final product. Indeed, a decisive impulse to superior biological and mechanical properties was given by the maintenance of bone-mimicking chemistry, nanostructure and unique 3-D hierarchical architecture, inherited by the original natural wood.

## Critical Aspects in 3D Gas-Solid Reactions

Heterogeneous gas-solid reactions have been largely investigated, particularly for application in the chemical and petroleum industries, and are fundamental phenomena involved in heterogeneous catalysis processes (Kreider and Lipiński, 2018; Groppi and Tronconi, 2000). However, differently from catalysis, where the solid phase plays the role of enhancer or modulator of the reaction kinetics but remains virtually unchanged during the whole process, here the solid is subjected to chemical changes with continuous alteration of the composition and structure so that the system is inherently unsteady with time. Generally, critical phenomena occurring in the course of gas-solid reaction systems can be resumed in: i) the adsorption of gaseous reactants on the solid surface; ii) the actual chemical reaction between the adsorbed gas and the solid surface; iii) the diffusion of gaseous reactants through the solid product formed at the surface to activate the heterogeneous reaction also in the solid bulk (Vinu, 2017; Xu et al., 2012).

However, such a scenario is further complicated when it comes to transform solids with 3D functional architecture rather than particles: in some cases, besides the achievement of successful phase transformation, a major purpose is also to retain all functionally valuable structural and mechanical features of the reacting solid. This aspect is exacerbated by the relatively low reactivity of inorganic solids at room temperature, that forces to adopt thermal treatments at conditions suitable to make effective the heterogeneous reaction process. In this respect, structural parameters such as porosity, specific surface area, pore size distribution, that can markedly affect the kinetic of the heterogeneous reactions and even their progress, can be subjected to important changes during the transformation process. The existence of all these concomitant effects render the understanding of the overall gas-solid 3D system and–consequently- the setup of gas-solid reactions yielding effective biomorphic transformation, a definitely non-routine task requiring originality and deep investigation of all the rate-controlling steps and their inter-relation.

**FIGURE 1:**
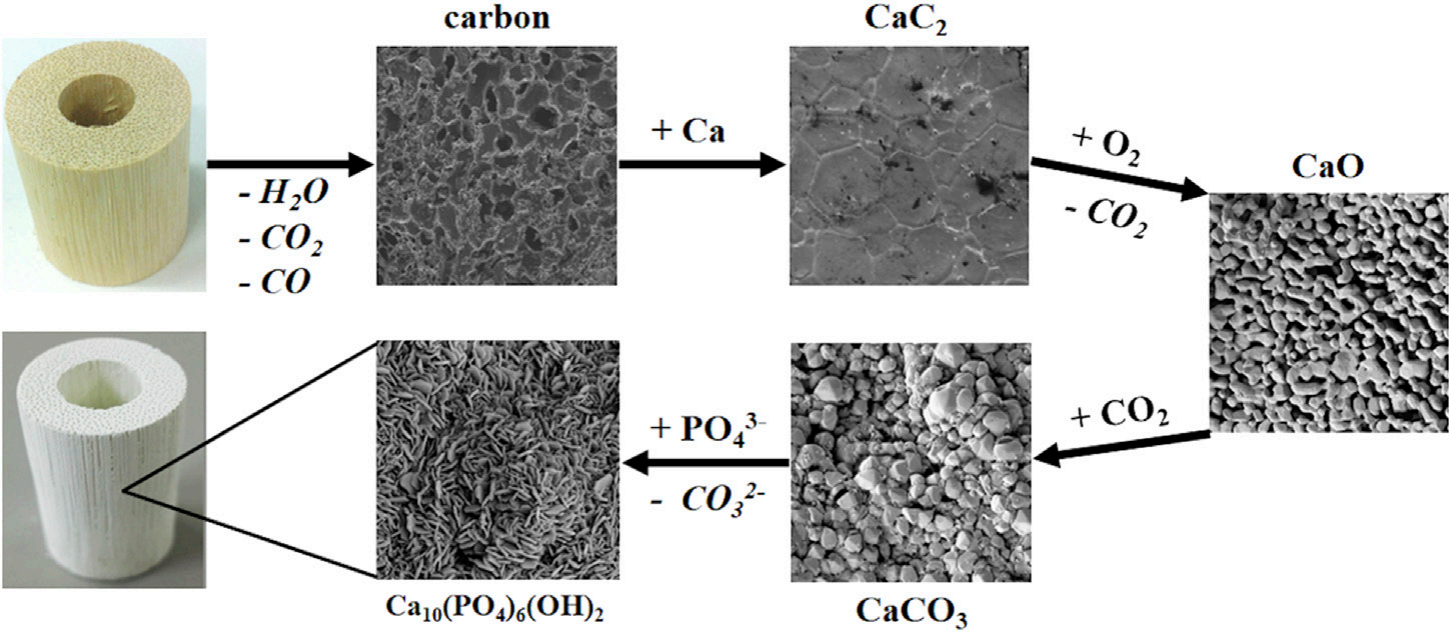
Scheme of the multi-step biomorphic transformation process (Images magnifications: carbon 5000X; calcium carbide, calcium oxide, calcium carbonate and hydroxyapatite 25000X).

The biomorphic transformation of rattan wood into a hierarchically organized scaffold addressing the regeneration of long, load-bearing bone segments, was obtained by a multi-step process (Figure 1) based on heterogeneous gas-solid reactions transforming the wood into a sequence of intermediate products, i.e., carbon, calcium carbide, calcium oxide and calcium carbonate, prior to the conclusive hydrothermal treatment transforming the calcium carbonate into the final product: a scaffold made of hydroxyapatite and tricalcium phosphate phases, both characterized by partial substitutions with Mg^2+^ and Sr^2+^ ions (Tampieri et al., 2019).

In the design of such a process, relevant aspects to be kept into strict consideration were identified as: i) the operating conditions (i.e., temperature and partial pressure of the reacting gas) that activate specific gas-solid reaction(s) yielding the nucleation of the new solid phase; ii) the presence of pores into the newly formed solid product favouring the penetration and diffusion of the gaseous reactant into the solid bulk; iii) the volume variation accompanying the formation of the new solid phase.

All these aspects are closely inter-related and greatly depend on the reaction kinetics. On one hand, the reaction conditions, basically related to the thermodynamics of the reacting system, should be chosen in order to achieve a reaction rate, effective for practical purposes. Generally, from a chemical perspective the use of higher temperatures, in appropriate ranges, enhances the reactivity and the reaction rate but, conversely, it can also induce adverse effects. For instance, temperature-induced grain growth can provoke reduction of intergranular porosity, thus limiting or even preventing the diffusion of gaseous reactants from the surface to the inner part of the reacting solid. Furthermore, the grain growth, yielding a decrease of the specific surface area and of active sites at the surface, can reduce the driving force for chemical transformation, so that the use of high temperature can induce various rate-limiting effects, penalizing the whole transformation process. Volume variations invariably occur during phase transformations, and these structural changes may represent a major issue affecting not only the integrity and mechanical stability of the solid reactant, but also its 3D architecture, including pore size distribution, hierarchical organization and interconnection. Changes in pore size distribution can help to accommodate deformation of the solid volume at the multi-scale during phase transformation, however these changes have to be controlled when the role of pore size distribution is functional.

## Towards Bioactive, Mechanically Competent 3D Nanostructured Ceramic Scaffolds

The understanding and consideration of all the above-described concomitant phenomena helped to design effective processes to transform a vegetal structure into a bioactive scaffold. In particular, starting from the rattan wood a major requirement for the final success was the maintenance, in all the intermediate products, of functionally-relevant multi-scale structure, adequate physical integrity and chemical reactivity sufficient to enable the subsequent transformation steps up to the final product.

To face critical stages of the multi-step biomorphic transformation process involving gas-solid reactions, we found that the use of minimal energy conditions was relevant to control the kinetics of the different reactions involved in the transformation process and the diffusive phenomena that allowed the reactions to proceed from the surface to the bulk. In turn, both these aspects are strongly inter-related with the compositional and microstructural evolution of the reacting solid, particularly relevant when high specific surface and the maintenance of interconnected multi-scale porosity facilitate the mass transfer and the progress of the heterogeneous reactions.

### Pyrolysis, Carburization and Oxidation of Natural Rattan Wood

The multi-step biomorphic transformation process started with the pyrolysis of the wood template, i.e., a thermal treatment carried out in oxygen-free atmosphere to transform the wood into inorganic carbon bodies suitable as template guiding the subsequent heterogeneous reactions. The pyrolysis process implied a huge mass loss and reduction of the original volume, due to the decomposition of the organic components, such as cellulose, hemicellulose and lignin, as well as water and other gaseous byproducts such as carbon dioxide and monoxide. In order to accomplish such a relevant volume variation and prevent structural and morphological deformations in the final carbon template, the pyrolysis process was carried out at very low heating/cooling rate (i.e., ∼1°C/min) (Tampieri et al., 2009). After pyrolysis, the first relevant step was the introduction of calcium in the reacting system through the formation of calcium carbide (CaC_2_) phase. Preliminary approaches consisted in the exposure of the carbon template to gaseous calcium in oxygen-free atmosphere, obtained by heating calcium granules above their boiling point (i.e., 1,484°C) (Tampieri et al., 2009). In spite the process was successful, when applied to carbon templates with large dimensions, the reaction was less effective, often leaving unreacted carbon in inner regions of the solid. Considering that the subsequent transformation step was the oxidation of CaC_2_ into calcium oxide (CaO) in air atmosphere, the thermal oxidation of the residual unreacted C resulted into formation of void regions, penalizing the mechanical integrity of the 3D product.

**FIGURE 2:**
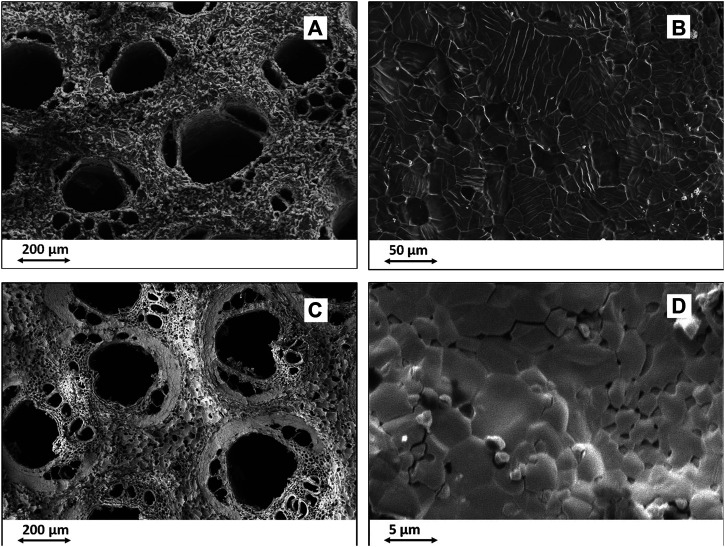
SEM images of biomorphic CaC_2_ obtained by heterogeneous Ca-C reaction. **(A,B)**: CaC_2_ obtained at ∼1,500°C; **(C,D)**: CaC_2_ obtained at T < 900°C. **(A,C)**: overall biomorphic microstructure; **(B,D)**: detail showing the grain size and morphology.

This finding shows how diffusive phenomena permitting the gas-solid interaction in the whole volume of the reacting template become progressively more critical as the template increases in size. To face this impasse, a more recent experiment was carried out by inducing the Ca-C chemical reaction at lower temperatures by decreasing the atmospheric pressure in the reaction chamber, thus obtaining a strong reduction of the boiling point of calcium (Tampieri et al., 2017). It was thus possible to conduct the whole carburization reaction at minimal energy conditions, suitable to activate the nucleation of CaC_2_ crystals but limiting their growth. This permitted to achieve greatly reduced grain size, from ∼100 μm down to ∼10 μm, and to retain substantial intergranular nano-porosity favouring the diffusion of the reacting calcium gas into the inner part of the C template, thus resulting in the complete conversion of C into CaC_2_. In this respect, Figures 2A,B show the microstructure of CaC_2_ obtained by Ca-C reaction carried out at ∼1,500°C, whereas Figures 2C,D report the microstructure of CaC2 formed at T below 900°C. To be noted the nanosized porosity in samples treated at lower temperature (Figure 2C in comparison with Figure 2A) and the much smaller particles obtained at lower temperatures (comparison between Figure 2D and Figure 2B).

**FIGURE 3:**
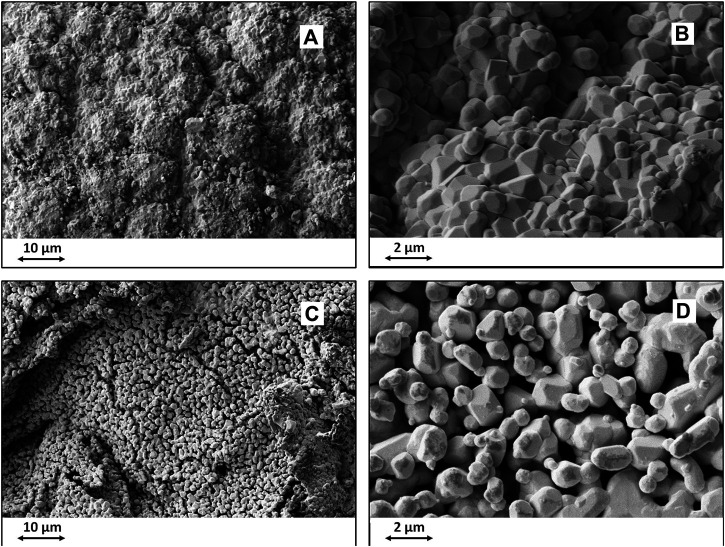
SEM images of biomorphic CaO obtained by oxidation of CaC_2_ precursor. **(A,B)**: CaO obtained by transformation of CaC_2_ synthesized at high temperature; **(C,D)**; CaO obtained by transformation of CaC_2_ synthesized at low temperature. **(A,C)**: overall biomorphic microstructure; **(B,D)**: detail showing the grain size and morphology.

To better accommodate structural inhomogeneity at the multi-scale, related to changes occurring in the crystal structure when converting C into CaC_2_, the reaction temperature was reached upon slow heating (i.e., below 10°C/min). The applied processing conditions permitted to obtain biomorphic CaC_2_ bodies retaining an interconnected network of intergranular nanopores that facilitated the diffusion of oxygen gas during the subsequent transformation step, aimed at the thermal oxidation of the CaC_2_ body into a biomorphic CaO (Figure 3).

Further retained up to the final scaffold, such a porous nanostructure also has invaluable utility in favouring the exchange of nutrients and autogenous growth factors when implanted *in vivo*, and improving the bio-resorption ability thanks to the higher specific surface area.

### Generation of Highly Reactive Calcium Carbonate Biomorphic Precursors

The subsequent step was the transformation of the obtained CaO into biomorphic CaCO_3_ bodies, intended as a precursor for the final product, i.e., the apatitic bone scaffold. The hydrothermal conversion of calcite into hydroxyapatite has been studied for decades (Verwilghen et al., 2009; Onoda and Yamazaki, 2016; Yoshimura et al., 2004); in the majority of cases the process was carried out on CaCO_3_ particles, granules, or even on corals, but never attempted to achieve a biomorphic hydroxyapatite body characterized by multi-scale hierarchy and organized porosity. Recent experiments to convert a wood into biomorphic calcium carbonate were carried out by heating a CaO template at 400°C under CO_2_ gas pressure (2.2 atm) or, differently, by heating a CaO template at 900°C in pressure-less CO_2_ atmosphere (Tampieri et al., 2009). In both cases the reaction occurred at the CaO surface but with very slow penetration rate into the template bulk, thus resulting in incomplete reaction overall. In the first attempt, the low temperature and low pressure adopted were ineffective to induce gas diffusion into the CaO body; on the other hand, when using high temperature treatment, the enhanced thermally-induced grain growth provoked the formation of a surface layer poorly permeable to further CO_2_ gas penetration, thus hampering the progress of the reaction towards the inner core of the bulk structure.

To solve the problem, a more recent study made use of a furnace capable of thermal treatments under gas pressure. CaO templates were heated under CO_2_ pressure (100 atm), thus reaching supercritical conditions that greatly increased the reactivity of CO_2_ and enabled nearly complete conversion of CaO into CaCO_3_ at T ∼800°C. In spite of this success, the obtained CaCO_3_ body could not be used for subsequent transformation into hydroxyapatite, as planned: in fact, the sample was subjected to spontaneous disintegration within short time after its achievement. Such an occurrence shows how chemical, physical and mechanical features are closely inter-related when the goal is to achieve a biomorphic transformation of large macroscopic samples, where indeed diffusivity phenomena become increasingly relevant. In fact, the disintegration was ascribed to residual stresses accumulated in the CaCO_3_ body as provoked by the thermally-induced grain growth. Therefore, even in supercritical conditions enhancing the reaction kinetics, it was not possible to prevent the CaCO_3_ grain growth. In a different approach, the CaO-CO_2_ reaction was further facilitated by introducing water vapour in the chamber to induce the formation of a thin aqueous film on the CaO surface during the process. Such a hydration layer could accelerate the CaO-CO_2_ reaction by inducing the formation of calcium hydroxide (Ca(OH)_2_) at the surface. Such an occurrence was previously reported as detrimental for the integrity of the solid CaO reactant, because the large volume change occurring during transformation of CaO to Ca(OH)_2_ provoked its prompt disintegration. However, in the presence of the aqueous layer, the reacting CO_2_ gas could dissolve thus creating an acidic aqueous environment rising the solubility of the newly formed Ca(OH)_2_ phase, thus making available free Ca^2+^ ions which, reacting with the CO_2_, formed the CaCO_3_ phase at a much lower temperature. The above mentioned adverse effects related to the formation of Ca(OH)_2_ could be avoided thanks to the use of high gas pressure in supercritical conditions which permitted the formation of Ca(OH)_2_ only as a transient phase. The use of high pressure was a key to achieve the penetration of the gaseous CO_2_ reactant in the inner regions of the CaO solid until complete transformation occurred.

The CaCO_3_ precursors with enhanced mechanical strength and smaller grain size obtained with this method (i.e., ∼1–2 μm vs 10–20 μm) was accompanied by enhanced intergranular porosity and higher specific surface area, thus increasing the chemical reactivity and ability to undergo dissolution-reprecipitation into hydroxyapatite during the final transformation step, as described in the following paragraph.

### Hydrothermal Reactions to Generate 3D Hydroxyapatite Scaffolds With Designed Crystal Structure and Nanotexture

Hydrothermal reactions can be defined as wet chemistry techniques making use of an aqueous solvent above its boiling temperature and pressure. Hydrothermal reactions are characterized by phenomena of chemical dissolution, nucleation and growth of inorganic crystals, that can be modulated by controlling fundamental parameters such as temperature/pressure, pH, and reaction time. In turn, the control of such parameters can yield enhanced control of the shape and morphology of the particles forming the product.

The thermodynamic driving force for hydrothermal reactions, i.e., the molar Gibbs energy, is expressed by the simple formula:

$$\Delta \text{G }=\;-\left(\text{RT/n}\right)\;\text{ln}\left({\text{A/K}}_{\text{sp}}\right)=\;-\left(\text{RT/n}\right)\;\text{ln}\left(\text{S}\right)$$ (1)

where ΔG is the molar Gibbs energy; R is the universal gas constant; T is the absolute temperature; n is the number of ions in the product molecule and S is the supersaturation degree that is defined by the ratio between the activity product of ion units (A) and the corresponding solubility product (K_sp_), at a given temperature. It is thus evident that the kinetics of hydrothermal processes are strongly related to the process temperature and, in a minor extent, to the supersaturation extent. As a general rule, high temperature leads to the formation of large and long fibres/particles, while at lower temperature small dimensions are preferred (Loo et al., 2008; Zhang and Vecchio, 2007; Jiang and Zhang, 2009). When experimental conditions are tuned to decrease the free energy variation, the reaction proceeds slower, thus nucleation of the product occurs at a reduced rate but this may result in drawbacks in terms of enhanced crystal growth. In this respect, the control of the saturation index, defined as SI = log (S), allows the fine tuning of the crystal size: when SI < 0, the solution is undersaturated and dissolution processes are favoured, if S = 0, the system is in equilibrium, while at SI > 0 crystallization is favoured, with higher nucleation rates when SI increases.

Another major parameter affecting hydrothermal processes is the pH. Particularly, when considering the specific case of CaCO_3_ conversion into hydroxyapatite (HA), the tuning of pH can modulate the distribution of various phosphate species (i.e., H_3_PO_4_, H_2_PO_4_^−^, HPO_4_^2−^, and PO_4_^3−^) characterized by different pH values at the equilibrium point. An increment of pH value shifts the phosphate species equilibrium from H_3_PO_4_ ➔ H_2_PO_4_^−^ ➔ HPO_4_^2−^ ➔ PO_4_^3−^, respectively, and increase the saturation index of HA according to the following equation (Viswanath and Ravishankar, 2008):

$$\text{Log}\left({\text{SI}}_{\text{HA}}\right)\;=\text{ 4 pH}-72\text{.}08$$ (2)

This means that SI > 1, a condition leading to HA nucleation and growth, can be reached at pH values >5. It was observed that, moving from acidic to alkaline conditions, the morphology of the newly formed HA particles changes from need-like or rod-like shape to smaller and more rounded particles (Earl et al., 2006; Sadat-Shojai et al., 2011; Salariana et al., 2008).

The above general concepts also apply to cases when wet synthesis processes carried out at low temperature or under hydrothermal reactions involve a solid reactant. In biomorphic transformations, the purpose is to achieve phase transformation in a solid while retaining its original microstructural details at the multi-scale. In this respect, the overall dissolution/reprecipitation kinetics should be tuned, not only to achieve products with the desired composition and nanostructure but also, and importantly, with the purpose to prevent the disruption of the final material, either due to excessive dissolution rates or structural deformation related to the growth of the new phase. In this respect, previous experiments carried out in wet conditions with a CaCO_3_ piece obtained by biomorphic transformation of rattan wood, confirmed that temperature is a very crucial parameter determining the reaction rate (Ruffini et al., 2013). In fact, even when working at quite low temperatures (i.e., 20 or 40°C), slightly acidic conditions provoked excessive dissolution rate, thus provoking the disruption of the material; at higher temperatures (i.e., up to 60°C) similar results were obtained also at more alkaline conditions.

Such a phase transformation resulted more effective when applying hydrothermal treatments in alkaline conditions to CaCO_3_ biomorphic precursors characterized by high reactivity (Tampieri et al., 2019). Such a process could fasten the transformation of CaCO_3_ into hydroxyapatite, characterized by lamellar nanostructure similar in morphology to the inorganic part of bone. This achievement was facilitated also by topotactic information given by the CaCO_3_ crystals, yielding the epitaxial growth of the new apatitic phase along specific crystal directions (Álvarez-Lloret et al., 2010).

**FIGURE 4:**
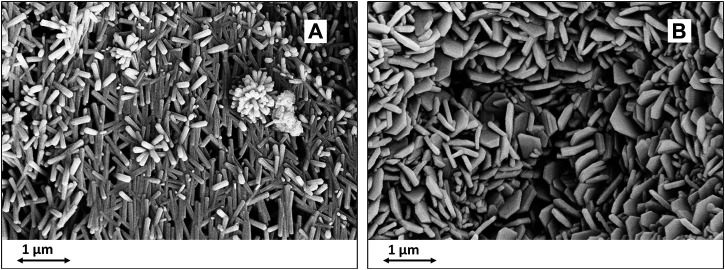
SEM images of nanostructured biomorphic scaffolds as obtained by different hydrothermal conditions. **(A)**: neutral pH; **(B)**: alkaline pH.

At this point, the development of specific nanostructures can be driven by tuning processing parameters such as pH, ionic strength and reaction time. During the process, self-assembling phenomena can lead to coalescence of the newly formed HA particles into nanosized superstructures with lamellar, petal-like or flower like morphologies (Ruffini et al., 2019). (Figure 4) The wet process yielding the final scaffold can be carried out under conditions permitting to modulate the composition of the apatitic phases at the atomic level, by activating diffusive and ion exchange phenomena driven by temperature and pH. In this respect, it is possible to induce the partial replacement of Ca^2+^ ions with foreign biologically-relevant ionic species, that alter the chemistry and crystallinity of the apatitic phase thus enhancing the bioactivity, particularly the capability of ion exchange in physiologic environment and the bio-resorbability. Relevant bioactive ions include Mg^2+^, Sr^2+^, CO_3_^2-^, SiO_4_^4-^, Zn^2+^ which are well known to enhance the osteogenic process and promoting the cross-talk between different cell lines active in bone regeneration such as osteoblasts, osteoclasts and endothelial cells (Montesi et al., 2017; Landi et al., 2008; Bigi et al., 1992). The ion exchange process can be performed by varying the parameters influencing the diffusion rate of doping ions, i.e., temperature and ion concentration, according to the Gibbs equation for ion exchange phenomena in a closed system (i.e., ΔG = RT ln(Q/K_q_), where Q is the reaction quotient and K_q_ is the standard equilibrium constant). In a previous study it was possible to obtain the partial replacement of Ca^2+^ ions with small amounts of Mg^2+^ and Sr^2+^ ions that are among the most relevant cell instructors to promote and sustain new bone formation and maturation (Tampieri et al., 2019; Galli et al., 2017; Saidak and Marie, 2012).

### Biologic Performance of Biomorphic Apatitic Scaffold

The relevance of the above approach resides in the outstanding results coming from various biological and mechanical tests. The unique composition and structure of the biomorphic scaffold were the source of great osteogenic ability in a bioreactor study: Tampieri et al., 2019, the biomorphic scaffold induced over-expression of various genes involved in osteogenesis, greatly enhanced in comparison with a sintered apatitic scaffold featuring similar porosity extent. This finding confirmed the great relevance of biomimetic scaffold composition and multi-scale structure, characterized by ability of ion exchange and bio-solubility, to act as instructors for cells, in comparison with sintered bodies that, in spite very good biocompatibility, are too chemically inert to enable bioactive interaction with cells. In the same study, the biomorphic scaffold also demonstrated ability to induce and guide the formation of new bone with organized osteon-like morphology, when subcutaneously implanted in rabbits. Such result is, as well, of great relevance because it demonstrates that topological information given by the biomimetic structural hierarchy is able to effectively guide the direct formation of mature and structurally organized tissue, even when the scaffold was implanted in ectopic site, i.e., free of any osteogenic endogenous signal, added cells or growth factors. It is worth to consider that, lacking such instructive information, osteoinductive ability is usually observed by preliminary formation of disordered woven bone and further occurrence of remodelling, that however can occur only in response to specific mechanical needs that usually do not occur in ectopic site.

From a mechanical perspective, the achievement of biomorphic structure with multi-scale hierarchy was relevant to yield damage-tolerant mechanical behaviour, similarly as occurs with the natural bone. This is a very unusual feature for pure ceramic materials that are overall recognized as brittle, a feature that greatly limit their application in load-bearing applications. Indeed, it was recently reported that, together with the multi-scale architecture of the natural construct from which they originate, biomorphic materials inherit also their vascularizing and mechanical performance so that, from a mechanical perspective, they can be considered as a new class of inorganic compounds (Sprio et al., 2020; Bigoni et al., 2020). Scaffolds with bone-like mechanical performance can mimic the behaviour of the natural bone under loading, thus being very promising as an instructor for the activation of cell mechanotransduction phenomena, particularly relevant in load-bearing bones. Such hypothesis was confirmed by a recent *in vivo* study, carried out by implanting the biomorphic scaffold in a critical segmental defect (2 cm) in sheep metatarsus, in comparison with allograft (Kon et al., 2021). Analysis of the explants after 6 months revealed effective osteogenesis, osteointegration and vascularization involving the whole scaffold volume, thus leading to regeneration of the whole segmental bone defect. Such phenomena were related to extensive bio-resorption of the scaffold, permitted by its biomimetic composition and nanostructure, in turn enabling physiologic metabolic activity by endogenous bone and endothelial cells so that extensive vascularization could also occur. This latter was also favoured by the unique 3D architecture of the scaffold which also yielded appropriate mechanical performance. In fact, mechanical analysis of the explants carried out by microhardness reported the recovery of bio-competent mechanical properties in the newly formed bone, as related to appropriate mineralization extent, whereas osteopenia at bone-scaffold interface was observed with allografts. Considering that such study was carried out in a large animal model by using a cell-free device without any added growth factors, these results confirm that the use of 3D biomorphic scaffolds recapitulating chemical composition, 3D hierarchical architecture and mechanical behavior of the natural bone tissue, is a promising way to achieve extensive regeneration of long load-bearing bone segments in real clinical scenarios.

## Conclusion: Perspectives for Future Applications of Biomorphic Materials

The mankind has always observed with curiosity and amazement the functional abilities of many living beings that populate the earth, air and water, dreaming one day of imitating them. In recent years, materials scientists have stepped up their efforts to investigate the structural mechanisms that determine such unique capabilities; however, establishing technological processes able to develop devices that replicate such mechanisms is still an unsolved challenge. In this regard, biomorphic transformation processes represent a very promising approach to achieve this goal, also in consideration of recent results showing the feasibility to obtain macroscopic constructs with controlled composition, nanostructure and 3D multi-scale architecture that synergistically generate outstanding and unusual properties. In particular, it has been shown that the application of heterogeneous chemical reactions in the 3D state allows to transform a natural wood into a ceramic bone scaffold with a hierarchically organized architecture, endowed with unpreceded biological and mechanical properties, which could one day allow to solve clinical cases of great relevance in regenerative medicine, but still unmet.

In a broader perspective, obtaining inorganic constructs with biomorphic structure could open the way, in the near future, to functional applications that are still unexplored as they are not feasible with current devices. In this regard, the study and application of biomorphic transformation processes are still at their infancy; however, given the presence of innumerable natural structures from which materials scientists can draw inspiration, it is foreseeable that the study of new processes based on heterogeneous chemistry in the 3D state might constitute a new area of research in materials science in the incoming years.
